# Particularities in the “Oldie but Goldie” Tc-99m DMSA Renography: A Retrospective Reference Centre Overview of 931 Children

**DOI:** 10.3390/diagnostics15081025

**Published:** 2025-04-17

**Authors:** Irena Cristina Grierosu, Iuliana Magdalena Starcea, Wael Jalloul, Maria Adriana Mocanu, Roxana Alexandra Bogos, Tudor Ilie Lazaruc, Madalina Andreea Beldie, Ruxandra Tibu, Teodor Marian Ionescu, Cati Raluca Stolniceanu, Brindusa Casiana Acsinte, Cipriana Stefanescu, Alexandra Saviuc, Vlad Ghizdovat

**Affiliations:** 1 Faculty of Medicine, “Grigore T. Popa” University of Medicine and Pharmacy, 700115 Iasi, Romania; 2 Nuclear Medicine Laboratory, “St. Spiridon” County Emergency Hospital, 700111 Iasi, Romania; 3 Department of Paediatric Nephrology, “St. Maria” Emergency Children Hospital, 700309 Iasi, Romania; 4 Paediatric Department, “St. Maria” Emergency Children Hospital, 700309 Iasi, Romania

**Keywords:** Tc-99m DMSA scan, paediatric renal malformations, differential renal function, chronic renal failure

## Abstract

**Background/Objectives**: The Tc-99m dimercaptosuccinic acid (DMSA) renal scan clearly images the renal cortex, highlighting functional tissue areas and indicating regions of renal scarring, infection, malformations, or other types of renal damage. To enhance the management of paediatric cases involving renal malformations and to reduce the incidence of chronic and progressive kidney diseases in “future adults”, our study aims to identify and categorise various renal anomalies. **Methods**: This has been achieved by analysing the Tc-99m DMSA renal scans of a large cohort of 931 children diagnosed with different renal pathologies. After interpreting the scans, we categorised the renal malformations and cortical modifications into four groups: kidney number anomalies, positional anomalies, structural anomalies, and shape anomalies. **Results**: There has been a notable increase in the demand for renal scintigraphy in recent years, rising from 82 cases in 2019 to 183 cases in 2024. Structural anomalies were the most common type of malformations (73% from all patients), featuring a significant variety of cortical modifications. In total, 98 cases (93% from kidney number anomalies and 10.5% from all children) were diagnosed with renal agenesis. Additionally, 30 children (3.2% from all patients) had positional anomalies, primarily ectopic kidneys, and 54 patients (5.8% from all cases) had shape malformations, especially fused kidneys. **Conclusions**: Combining the Tc-99m DMSA renal scan with ultrasound provides a more reliable diagnosis of paediatric renal progressive diseases. A more accurate diagnosis allows for quicker treatment and prevention of potential complications, ultimately improving the quality of life and decreasing hospital costs of paediatric patients becoming adults.

## 1. Introduction

A variety of radiopharmaceuticals is used for evaluating renal function, including Tc-99m diethylenetriaminepentaacetic acid (DTPA), Tc-99m dimercaptosuccinic acid (DMSA), and Tc-99m mercaptoacetyl triglycine (MAG3).

While Tc-99m DTPA and Tc-99m MAG3 function as glomerular filtration agents, Tc-99m DMSA is a tubular secreted radiopharmaceutical primarily used to assess the structure of the renal cortex [1,2].

**Figure 1:**
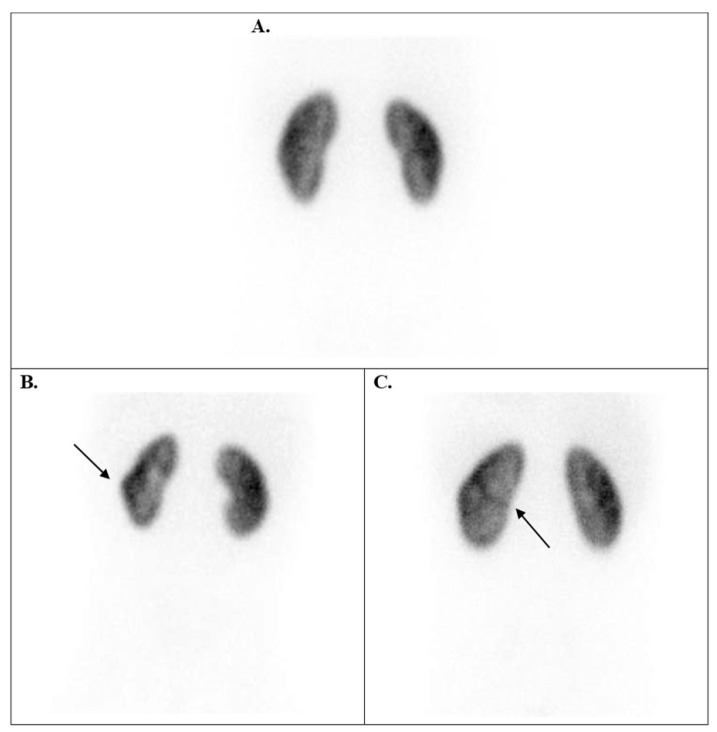
Tc-99m DMSA posterior planar images of normal variants. (**A**) Relatively homogeneous radiotracer uptake in the renal parenchyma in a 7-year-old boy with grade I HN. (**B**) A normal image of a 1-year-old boy with grade I–II left HN (the left upper pole appears flat due to spleen compression). (**C**) A 12-year-old boy has a left kidney with a double collecting system, typically showing a sharp line of demarcation between the poles, along with grade III left HN. HN = hydronephrosis.

Knowing that Tc-99m DMSA does not significantly enter the renal medulla or pass into the renal pelvis or ureters, this selectivity allows the Tc-99m DMSA renal scan to provide a clear image of the renal cortex, highlighting areas of functional tissue. The radiotracer uptake in normal kidney images appears homogeneous and maintains a regular shape, and the cortex is uninterrupted (Figure 1).

Areas with reduced uptake may indicate renal scarring, infection, or other types of kidney damage [3]. In this context, the main indications of a paediatric Tc-99m DMSA scan are as follows [4,5,6]: diagnosis of acute pyelonephritis, identification of renal scars after repeated urinary infections at least six months following an acute urinary tract infection (UTI), assessment of parenchymal damage following kidney trauma, detection of renal dysplasia, confirmation of non-functional multicystic dysplastic kidney, and assessment of ectopic kidneys and renal structural anomalies (renal duplication, horseshoe kidney, etc.). This nuclear medicine technique is also beneficial in determining differential renal function (DRF) in certain pathologies, such as ectopic or dysplastic kidneys, and before performing a nephrectomy on non-functioning or poorly functioning kidneys or a pole in a duplex kidney [7]. Moreover, in patients with an allergy to iodinated CT contrast and when MRI is unavailable or contraindicated, Tc-99m DMSA scintigraphy represents an option for evaluating renal parenchyma [6]. Another aspect is that in medical centres where anaesthesia for children cannot be administered due to a shortage of specialised practitioners, legal issues, or technical challenges, performing CT and MRI scans can be particularly challenging. One major concern is the movement artefacts that occur during these examinations. However, using Tc-99m DMSA scanning can provide a solution, as the image processing software can correct these movement artefacts.

Our study aims to enhance the management of paediatric cases with renal anomalies to reduce the risk of chronic and progressive kidney diseases, as well as renal-induced high blood pressure in “future adults”. In correlation with the indications for renal scintigraphy, we will focus on identifying and categorising various renal cortex anomalies. This will be achieved by analysing Tc-99m DMSA renal scans from a large group of children in the Northeastern region of Romania.

## 2. Materials and Methods

Our retrospective cohort study included 931 children under 18 years old (ranging from 2 months to 17 years) with various renal pathologies. These cases were referred by the Paediatric Hospital of Iasi, Romania, to the Nuclear Medicine Laboratory at the County Emergency University Hospital in Iasi, Romania, to undergo Tc-99m DMSA scintigraphy between January 2003 and January 2025. Patients suspected of having kidney scars presented with symptoms of urinary infections, which were confirmed by blood and urinary tests. The other categories of renal anomalies were discovered incidentally through ultrasound or due to lumbar pain. The selected children were from the Northeastern Region of Romania, which, as of 2019, comprised six counties and a population of 3.199 million people. It is worth noting that the Nuclear Medicine Laboratory at the County Emergency University Hospital and the Paediatric Hospital in Iasi are the reference centres that manage and investigate nephrology paediatric patients in this region.

All phases of the process were conducted in accordance with laboratory guidelines, which align with the published practice guidelines of the Society of Nuclear medicine and Molecular Imaging (SNMMI) procedure standard/the European Association of Nuclear Medicine (EANM) [6]. Moreover, as the nuclear medicine laboratory is part of a university hospital, the mothers of the children provided their informed consent for the potential use of their children’s medical data for research prior to each examination. Since the study was anonymous and retrospective, no further ethical approval was needed.

All patients received a paediatric dose of Tc-99m DMSA according to the guidelines, and the European Dose Card was used to determine the administered activity of the radiopharmaceutical [5]. Two h and 30 min later, static images (anterior, posterior, and left and right oblique posterior) were performed using a dual-detector Gamma camera, for a total time of approximately 15 min. Furthermore, single-photon emission computed tomography (SPECT) was performed when more fields of view were required for precise localisation and renal parenchymal characterisation.

**Figure 2:**
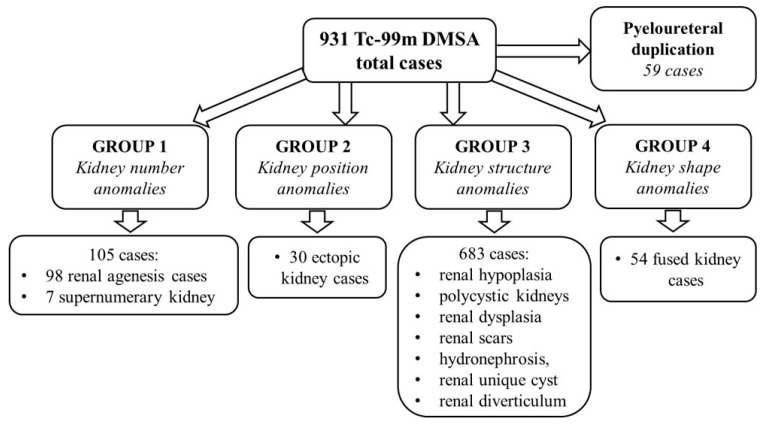
Classification of the renal malformations and cortical modifications into four groups. The 59 patients with pyeloureteral duplication were not included in our renal parenchyma anomalies-based classification.

Two nuclear medicine physicians analysed the total number of scans. To resolve a possible disagreement, a third nuclear medicine physician was consulted. Taking into account the radiotracer distribution, the renal parenchyma malformations and cortical modifications have been categorised into four groups as follows: Group 1: *kidney number anomalies* (renal agenesis, supernumerary kidney), Group 2: *positional anomalies* (ectopic kidney), Group 3: *structural anomalies* (renal scars post UTIs, renal hypoplasia, renal dysplasia, hydronephrosis (HN), polycystic kidneys, renal cyst, renal diverticulum), Group 4: *shape anomalies* (fused kidney) (Figure 2).

Considering that Tc-99m DMSA is taken up by the renal cortex to provide images of the renal parenchyma, our classification did not include the 59 patients with pyeloureteral duplication (6.3% from all the patients), which cannot be properly detected by this imaging technique.

## 3. Results

**Figure 3:**
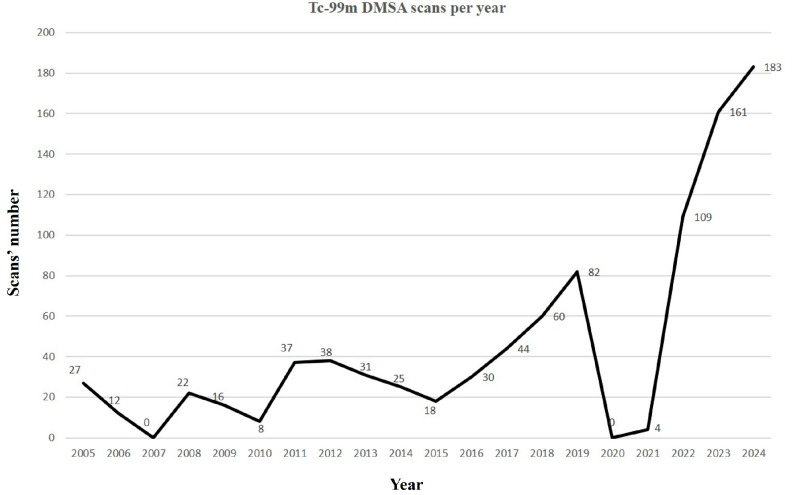
The number of Tc-99m DMSA paediatric cases per year. There was a nationwide radiotracer acquisition shortage in 2007 and 2008, and then, in the two years between 2020 and 2021, we only counted four cases because of the COVID-19 pandemic.

We noticed that the number of Tc-99m DMSA scans conducted in our department has changed over the years. Twenty years ago, there were only a few dozen cases, but in recent years, there has been a significant increase in the demand for renal scintigraphy. If in the first half of our study, the annual frequency was 19.5 cases, in the second half, it increased to 65.1 cases per year due to more findings of renal anomalies by antenatal ultrasound. It is essential to mention that there have been two periods when Tc-99m DMSA scans could not be performed. In 2007–2008, there was a shortage of DMSA. Furthermore, due to the COVID-19 pandemic, hospital restrictions and a decrease in paediatric consultations, only four cases were conducted between 2020 and 2021 (Figure 3).

**Figure 4:**
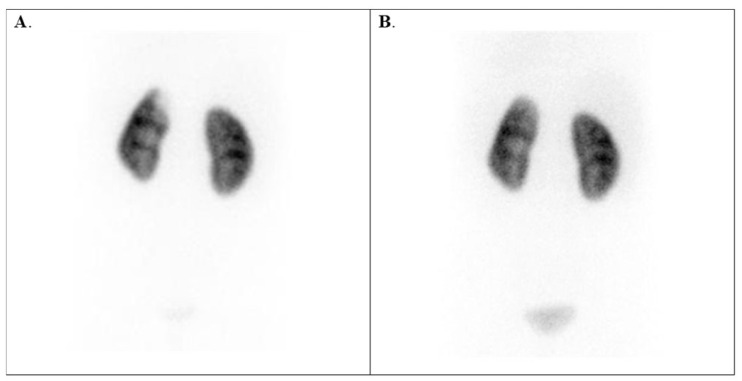
Tc-99m DMSA scan of a 9-year-old girl diagnosed with SARS-CoV-2 infection. (**A**) upper left polar renal infarction after SARS-CoV-2 infection. (**B**) Follow-up scintigraphy performed 2 years later shows the healed cortical function in the upper left pole.

Additionally, the pandemic has led to the emergence of new renal diseases. We recorded a unique case with SARS-CoV-2 infection, illustrated in Figure 4.

It is also worth highlighting the increase in renal scan requests from paediatric nephrology specialists in recent years, reaching the largest number of patients of 183 recorded children in 2024.

**Figure 5:**
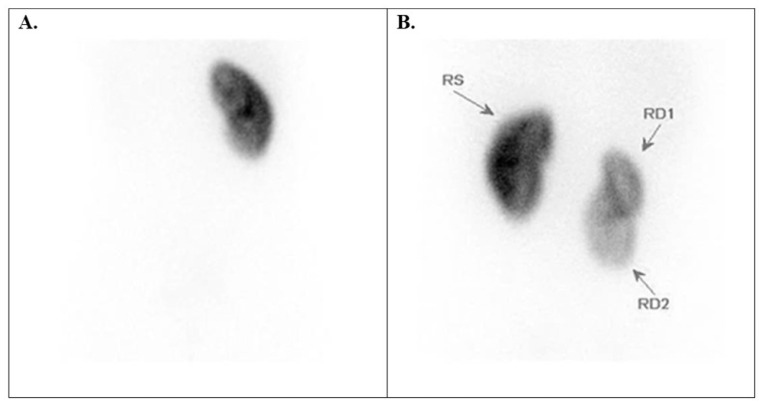
Tc-99m DMSA scans of kidney number anomalies cases. (**A**) left renal agenesia in a 16-year-old boy, and (**B**) a rare case of 3 kidneys (two right-sided) in a 14-year-old girl. RS = left kidney, RD = right kidney.

Children with *kidney number anomalies* (group 1) accounted for 11.3% (105 cases) of all included patients (Figure 2). The majority of these cases (98 cases) involved renal agenesis, all of which exhibited the unilateral agenesis type. In our retrospective study, boys were more affected than girls (56.1%), and left agenesis was predominant in 61 of the cases (62.2%) (Figure 5A).

In the same group, we counted only seven cases of supernumerary kidneys (0.75% of all patients) (Figure 5B).

**Figure 6:**
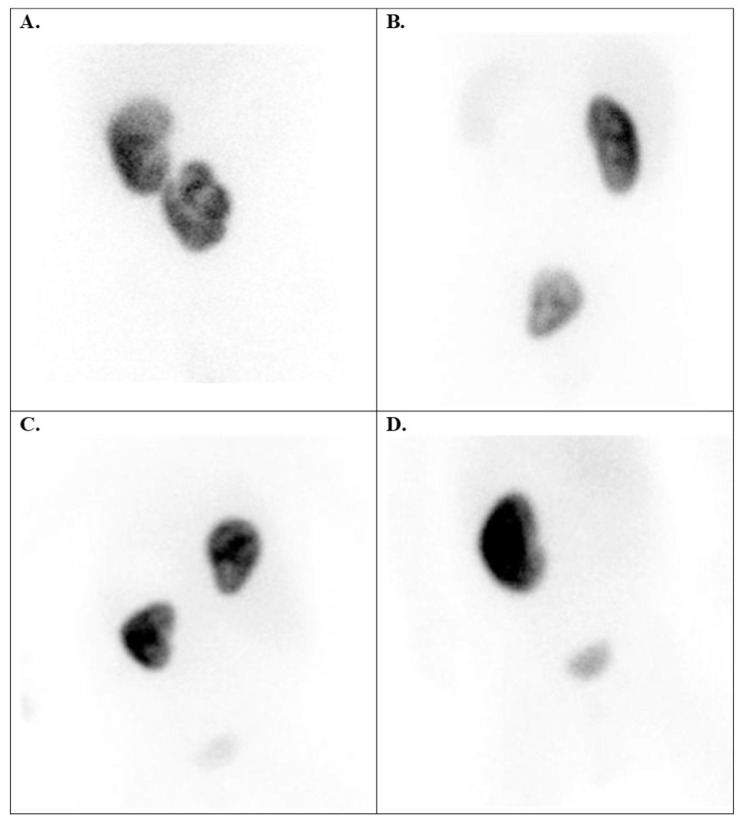
Tc-99m DMSA scans with kidney position anomalies. (**A**) Right-sided crossed kidney ectopy without fusion in a 13-year-old girl (confirmed with CT images). (**B**) Pelvic ectopic kidney in a 10-year-old girl, DRF: 43.3% left (ectopic) kidney and 56.7% right kidney. (**C**) A rare case of an ectopic right kidney in the right part of the chest in a 6-month-old girl with dextrocardia. (**D**) A hypoplastic paravesical (pelvic) kidney in a 5-month-old boy identified by renal scintigraphy (the ultrasound diagnosis prior to scintigraphy indicated right renal agenesis). DRF = differential renal function.

Thirty cases of ectopic kidneys were observed in patients with *kidney position anomalies* (group 2), with boys accounting for 62% of the cases (Figure 2). Among these cases, there were 18 instances of contralateral kidneys, such as cross-renal ectopia (with or without fusion) (Figure 6A), with the majority being left ectopia (61.1%).

Additionally, there were nine cases of pelvic kidney (Figure 6B,D), two cases of iliac kidney, and one case of ectopic thoracic kidney (Figure 6C).

**Figure 7:**
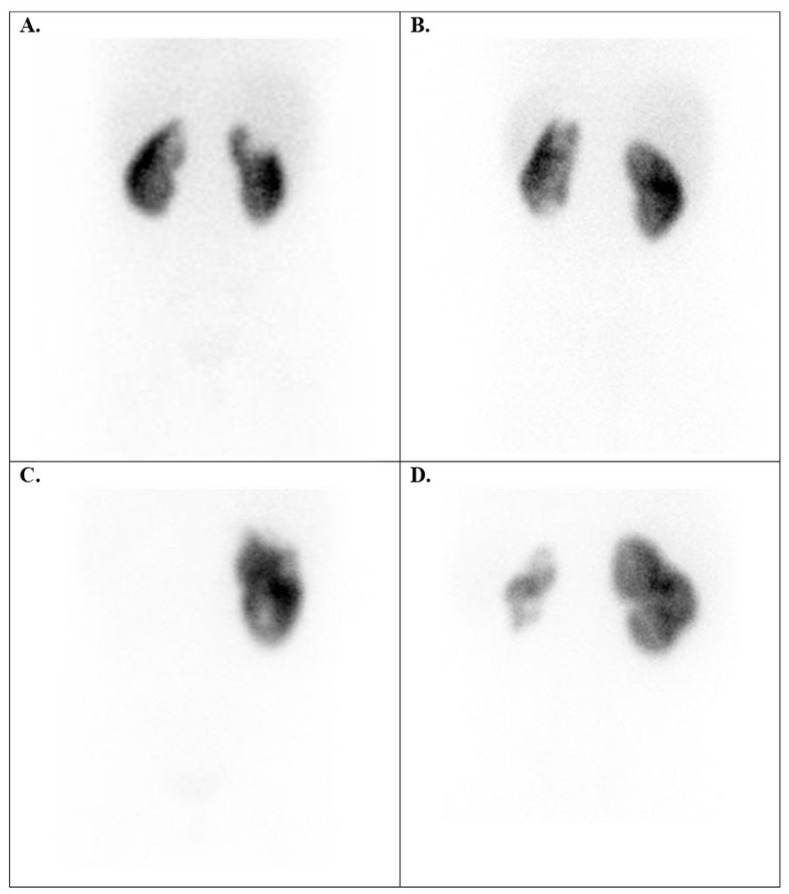
Tc-99m DMSA images of acute and chronic urinary tract infection cases. (**A**) Acute pyelonephritis in a 6-year-old girl (right upper pole defect in the cortex); (**B**) recurrent UTI episodes in a 9-year-old girl (left renal scars); (**C**) renal scars in a 4-year-old boy with antecedents of UTIs, VUR, and left nephrectomy; (**D**) hypoplastic left kidney and bilateral renal scars in a 12-year-old boy diagnosed with bilateral VUR, recurrent UTI, and right grade II HN. UTI = urinary tract infection, VUR = vesicoureteral reflux.

A total of 683 children were referred to our laboratory for a Tc-99m DMSA scan to assess the *kidney structure anomalies* (group 3), especially renal scars following episodes of UTI and vesicoureteral reflux (VUR) (Figure 7).

**Figure 8:**
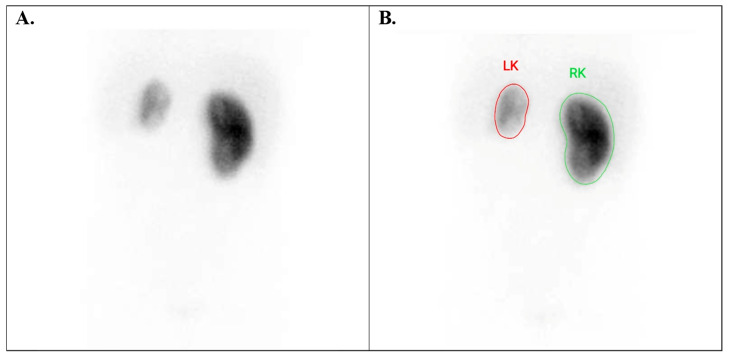
Tc-99m DMSA images renal hypoplasia. (**A**) A 7-year-old boy with left renal hypoplasia and (**B**) DRF calculation (16% LK and 84% RK) in the same patient. DRF = differential renal function, LK = left kidney, RK = right kidney.

**Figure 9:**
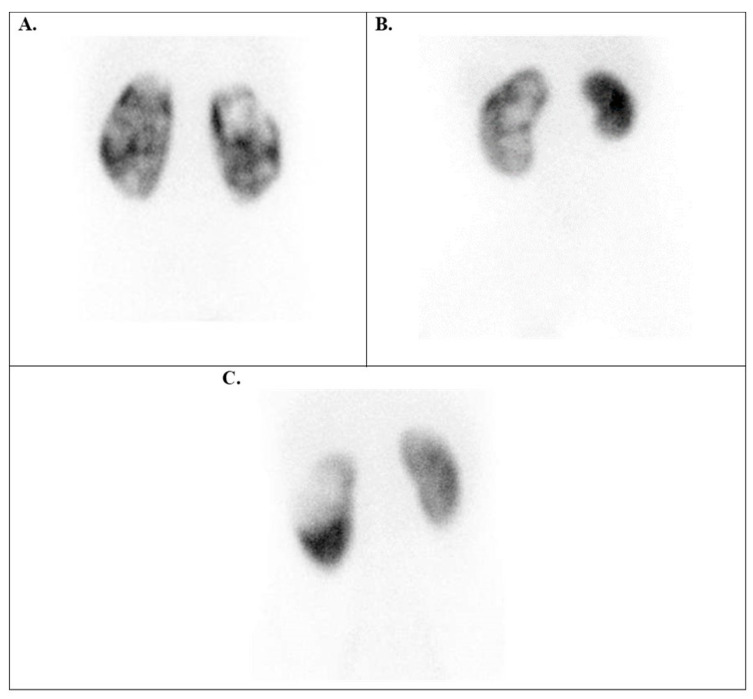
Tc-99m DMSA scans with kidney structure anomalies. (**A**) Autosomal dominant polycystic kidney disease in a 7-year-old boy diagnosed with bilateral grade III HN and secondary arterial hypertension. (**B**) Left grade IV HN in a 9-month-old boy (DRF: 54.3% left and 45.7% right kidney). (**C**) Right calyceal diverticulum in a 16-year-old girl (diagnosed with CT and confirmed by renal scintigraphy). HN = hydronephrosis, DRF = differential renal function.

Moreover, renal hypoplasia (Figure 8), polycystic kidneys (Figure 9A), renal dysplasia, hydronephrosis (Figure 9B), renal cyst, and renal diverticulum (Figure 9C) represented the most common indication for evaluating cortex function or damage using this type of imaging technique.

**Figure 10:**
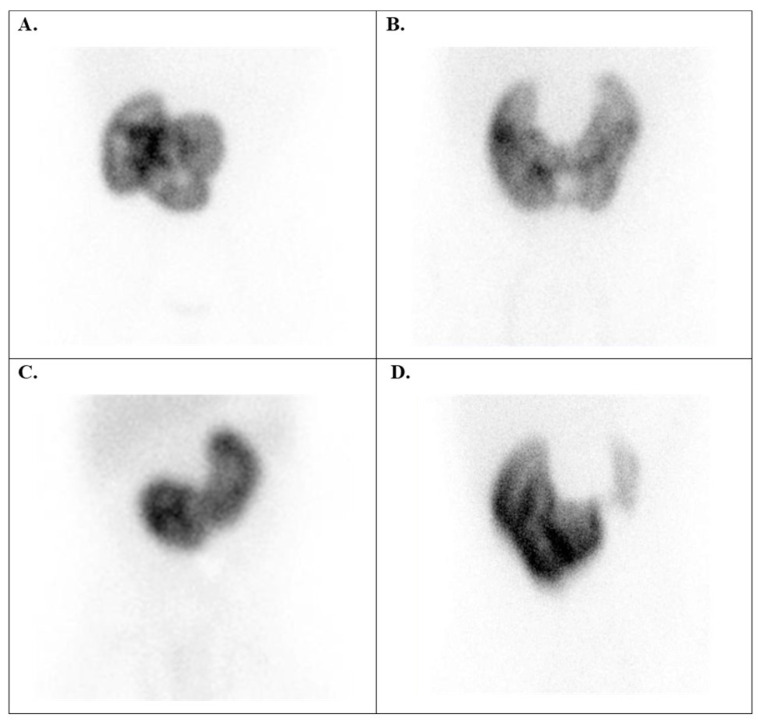
Tc-99m DMSA scans with kidney shape anomalies. (**A**) Fused kidneys (lump kidney) in 7-year-old boy (prior to scintigraphy, ultrasonography described a left renal agenesis and possible right ureteral duplication). (**B**) Horseshoe kidney in a 9-year-old girl patient. (**C**) Left lateral fused kidneys (L-shaped ectopia) in a 1-year-old boy (prior to scintigraphy, ultrasonography described an ectopic kidney). (**D**) A rare case of supernumerary kidney in conjunction with horseshoe anomaly in a 15-year-old boy.

Patients with *kidney shape anomalies* (group 4) consisted of 54 cases (5.8% of all patients) of fused kidneys, including horseshoe kidneys with parenchymal or fibrous isthmus, L-shaped ectopy, and lump kidney (Figure 10). Furthermore, 70.4% of the affected children were boys.

## 4. Discussion

### 4.1. Congenital Anomalies of the Kidney and Urinary Tract

An estimated 4 to 60 birth defects per 10,000 live births are caused by congenital anomalies of the kidney and urinary tract (CAKUT), which account for 20–50% of birth defects in humans [8]. Paediatric nephrologists, physicians, and/or urologists frequently refer newborns and infants with CAKUT for evaluation after subjecting them to radiologic and laboratory studies. Apart from determining the direct consequences of CAKUT, managing clinicians are frequently requested to forecast kidney outcomes in the long run and to pinpoint risk mitigation strategies [8].

One of the most severe health issues that children with CAKUT frequently face is chronic kidney disease (CKD) [9]. CKD is a chronic progressive condition affecting the kidneys and is becoming increasingly common [10]. In children, it can progress to end-stage kidney disease (ESKD), requiring dialysis or kidney transplantation [11]. A number of other chronic conditions, such as hypertension, cardiovascular disease, growth difficulties, electrolyte abnormalities, and metabolic bone disease, can also affect patients with CKD [12]. These children are also susceptible to sudden declines in health brought on by infections, dehydration, and adverse drug reactions [11,13]. Consequently, it is still unknown how CKD children will fare and what kinds of resources they will need in the hospital. For adults with CKD in the United States, Medicare spends more than $50 billion annually; however, comparable data on national healthcare costs for paediatric CKD are not available [14,15]. Prior research has observed that families with a child diagnosed with CKD face significant financial strains [16,17]. Because these children’s conditions are long-term and require specialised care, hospitalisation costs and management are a growing concern in the healthcare field. An appropriate diet, along with a proper and early diagnosis, could enhance these patients’ quality of life. Simultaneously, it might lower medical expenses in the latter phases of kidney dialysis or transplantation.

### 4.2. Paediatric Tc-99m DMSA Renography

Tc-99m DMSA renography is a safe and simple-to-perform imaging technique with high diagnostic sensitivity. Due to the reduced radiation dose injected into children, half-life of six hours, and gamma-ray emission, Tc-99m DMSA is considered the ideal radiopharmaceutical, indicated for diagnostics and follow-up of DRF in paediatric morphological renal pathologies, as well as for assessing long-term renal function outcomes in this category of patients [18]. Furthermore, renography is considered minimally invasive due to its lower radiation compared to intravenous urography (UVI) or UVI-CT [19]. This technique has the advantage of being able to perform multiple scans/views with a single injection and without exposing the patient to additional radiation. It also provides 3D tomographic images—SPECT. Moreover, the SPECT/low-dose CT in children is indicated because utilising attenuation correction will produce an accurate split renal function calculated by geometric mean [20]. Thus, the radioisotopic renogram is recommended in the diagnostic algorithm for patients, regardless of age, who have renal malformations detected by ultrasonography or persistent urinary tract infections. Additionally, increased findings of renal anomalies through ultrasound, which became mandatory during the antenatal and postnatal periods, along with improvements in awareness, have led to a greater demand for Tc-99m DMSA scans.

### 4.3. Tc-99m DMSA Renal Uptake Mechanism

**Figure 11:**
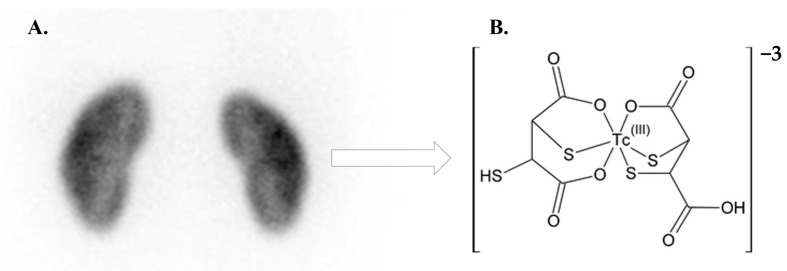
Tc-99m DMSA renal scintigraphy. (**A**) normal cortical Tc-99m DMSA cortical uptake, and (**B**) Tc-99m DMSA chemical formula.

After being administered intravenously, the trivalent Tc-99m DMSA (Figure 11) [21] is mainly transported to the kidneys by binding to plasma proteins. Within an hour after injection, the renal cortex takes up about 40% of the Tc-99m DMSA; the remaining portion is gradually eliminated through the urine over the next 24 h [22].

There is still much to learn about the precise uptake mechanism, which is not entirely understood.

The first theory suggests that Tc-99m DMSA enters proximal tubule cells through active transport and binds to sulfhydryl groups, possibly mediated by specific transport proteins [23]. According to Burckhardt et al. [24], the basolateral uptake of the radiotracer from peritubular capillaries into proximal tubule cells is facilitated by the sodium-dependent dicarboxylate transporter (NaDC-3). Afterwards, part of Tc-99m DMSA’s renal uptake is attributed to its reabsorption from the glomerular filtrate.

An alternative hypothesis regarding this radiotracer uptake mechanism is that the Tc-99m DMSA bound to α1-microglobulin is subsequently endocytosed by megalin/cubilin, which accumulates the Tc-99m DMSA protein complex in the kidneys. Under these circumstances, renal accumulation of Tc-99m DMSA is dependent upon the effectiveness of the megalin/cubilin receptor, which is a marker of proximal tubule endocytic activity [25]. Then, several physiological factors, including renal blood flow, glomerular filtration, and proximal tubule receptor-mediated endocytosis, undoubtedly influence the renal cortex’s ability to take up Tc-99m DMSA [26].

According to a 2021 study [27], the organic anion transporter is linked to the transport of radiotracer in renal proximal tubular epithelial cells. The basolateral membrane of the renal proximal tubule cells is usually where these transporters, primarily human organic anion transporter 3 (OAT3), are expressed. Afterwards, these anion transporters facilitate the tubular cells’ uptake of Tc-99m DMSA from the bloodstream.

### 4.4. Paediatric Tc-99m DMSA Renography Clinical Use and Indications

Our 22 years of experience in renal Tc-99m DMSA imaging, including 931 paediatric scans, showed an increase in the use of this technique in the last five to six years, with the number of scans rising from a few dozen to more than one hundred per year. However, due to the COVID-19 pandemic in 2020–2021, it was effectively impossible to perform the scans during that period (Figure 3).

In this paper, we presented 19 images of kidney anomalies and malformations visualised by Tc-99m DMSA in children, highlighting the most illustrative cases. Starting with the first group of patients presenting kidney number anomalies, renal agenesis was the most common malformation. It is defined as a congenital condition in which one or both kidneys fail to develop during foetal growth. Additionally, unilateral renal agenesis is classified as an orphan disease with a neonatal prevalence of around 1/2000 births [28]. This malformation is rare, but it significantly impacts foetal development and long-term health outcomes (Figure 5B).

Children with renal position anomalies had principally ectopic kidneys. These kidneys may be found anywhere along their usual path to the upper abdomen (pelvic, iliac, abdominal, and thoracic). A kidney can cross over to the same side of the body (cross renal ectopia); then, the two kidneys often grow together and possibly become fused. In accordance with Blickman et al. [29], our study showed that renal ectopia occurs more often in boys. Moreover, these scientists demonstrated that it is the most common type of renal malformation, affecting 1 in 500 newborns. In cases of a kidney with an abnormal position, assessing the DRF can be challenging due to the rotated and anterior position of the organ. In such situations, quantifying the DRF as a geometric mean between the anterior and posterior uptake of the radiotracer can be very helpful (Figure 8B).

The main indications for evaluating kidney structure anomalies include renal hypoplasia, polycystic kidneys, renal dysplasia, renal scars, HN, renal cysts, and renal diverticulum. Sometimes, distinguishing between polycystic kidneys and high-grade HN can be difficult without additional information or correlation with other structural images, such as ultrasound (Figure 9B,C). Additionally, renal scars are considered the most common renal structure abnormalities. They can be identified six months after episodes of acute pyelonephritis or in relation to chronic renal tract infections. Our results demonstrated that renal scars are more common in girls, while renal malformations occur more frequently in boys. Furthermore, the left side appears to be more affected. The data resulted in our statistics aligning with those published in the specialised literature [29,30]. However, in our study, kidney hypoplasia was the most prevalent renal anomaly.

Among kidney shape anomalies, fused kidneys, particularly different types of horseshoe kidneys, are the most common malformations. According to Schiappacasse et al. [30]., the incidence of horseshoe kidneys is about 1 in 500. Moreover, our study matches their findings, indicating a male preponderance of 2:1.

### 4.5. Paediatric Tc-99m DMSA Renography Limitations

It is crucial to consider various limitations while analysing the findings of our current study. The movement artefact was the most challenging aspect of image acquisition, making the SPECT and pinhole imaging difficult to perform, especially in children between a few months and 3 years old. We encountered this condition in 38 children, representing about 4% of the total number of patients. In these cases, even the image processing software was not sufficient to correct these movement artefacts. To address this issue, anaesthesia was utilised from 2003 to 2016. Then, due to a shortage of specialised practitioners in paediatric anaesthesia, legal issues, and technical challenges, from 2017 to 2019, a sedative syrup was used as a replacement for anaesthesia. Afterwards, starting in 2019, we implemented an animated movie visualisation protocol instead of medication to reduce the children’s anxiety and movement artefacts.

Another limitation of the study is that our nuclear medicine laboratory is located in a different hospital than the children’s hospital. As a result, we did not have access to pertinent demographic data needed to study and analyse the correlations between renal abnormalities and the patients’ demographic information.

## 5. Conclusions

The Tc-99m DMSA scan is a non-invasive technique that provides accurate structural and functional information about kidney malformations and urinary tract infections in children, holding an essential position in the diagnostic algorithm for kidney anomalies. When combined with prenatal or postnatal ultrasound and a well-defined diagnostic algorithm, renal scintigraphy leads to a more reliable and prompt diagnosis of paediatric renal progressive diseases. Thus, the sooner and more accurate the diagnosis, the faster we can treat and prevent any possible complications, leading to healthier adults in the future. Ultimately, the goal is to reduce the socio-economic impact of chronic kidney disease and improve the quality of life for these patients.

## Data Availability

The data presented in this study are available on request from the corresponding author.

## Abbreviations

Tc-99m diethylenetriaminepentaacetic acid (DTPA), Tc-99m dimercaptosuccinic acid (DMSA), Tc-99m mercaptoacetyl triglycine (MAG3), urinary tract infection (UTI), differential renal function (DRF), Society of Nuclear Medicine and Molecular Imaging (SNMMI), European Association of Nuclear Medicine (EANM), single-photon emission computed tomography (SPECT), hydronephrosis (HN), vesicoureteral reflux (VUR), congenital anomalies of the kidney and urinary tract (CAKUT), chronic kidney disease (CKD), end-stage kidney disease (ESKD), intravenous urography (UVI), sodium-dependent dicarboxylate transporter (NaDC-3), organic anion transporter 3 (OAT3), chronic renal failure (CRF), high blood pressure (HBP).
